# Supplementary material for: Observation of 2D-magnesium-intercalated gallium nitride superlattices
Source: Nature. 2024 Jun 5;631(8019):67–72. doi: 10.1038/s41586-024-07513-x (PMC11222157; doi:10.1038/s41586-024-07513-x)
Supplement: Supplementary file 1 — This file contains Supplementary Notes 1–4, Supplementary Figs 1–3 and Supplementary Table 1. [file 41586_2024_7513_MOESM1_ESM.pdf]

nature portfolio

https://doi.org/10.1038/s41586-024-07513-x

Supplementary information

Observation of 2D-magnesium-intercalated gallium nitride superlattices

In the format provided by the
authors and unedited

Supplementary Information for

Observation of 2D-magnesium-intercalated gallium nitride superlattices

Jia Wang*, Wentao Cai, Weifang Lu, Shun Lu, Emi Kano, Verdad C. Agulto, Biplab Sarkar, Hirotaka Watanabe, Nobuyuki Ikarashi, Toshiyuki Iwamoto, Makoto Nakajima, Yoshio Honda, Hiroshi Amano*

[1] *Institute for Advanced Research, Nagoya University; Nagoya, 464-8601, Japan.*

[2] *Institute of Materials and Systems for Sustainability, Nagoya University; Nagoya, 464-8601, Japan.*

[3] *Department of Materials Science and Engineering, Meijo University; Nagoya, 468-8502, Japan.*

[4] *Department of Electronics Engineering, Nagoya University; Nagoya, 464-8603, Japan.*

[5] *Institute of Laser Engineering, Osaka University; Osaka, 565-0871, Japan.*

*To whom correspondence should be addressed.

Emails: wang@nagoya-u.jp; amano@nuee.nagoya-u.ac.jp

Table of Contents

**Supplementary Note 1**	Changes in the valence band structure of strained GaN
**Supplementary Note 2**	Cross-sectional STEM observation of the MiGs morphologies on (0001) GaN
**Supplementary Note 3**	Constraint of lattice swelling in interstitial intercalation
**Supplementary Note 4**	Investigation of the formation and development of MiGs in p-GaN by SIMS
**Supplementary Fig. S1**	Atomic arrangement around a pair of 2D-Mg_i sheets
**Supplementary Fig. S2**	Compressive elastic strain in GaN layers intercalated with Mg sheets
**Supplementary Fig. S3**	Backside SIMS profiling of the p-type GaN after metallic Mg annealing and acid clean
**Supplementary Table 1**	Maximum coverage of Mg-intercalated GaN at different temperatures

Supplementary Note 1. Changes in the valence band structure of strained GaN

We performed *ab initio* calculation to investigate how uniaxial compressive strain affects the bandgap and internal parameter. As shown in Extended Data Fig. 3a, the relationship between the uniaxial compressive strain and the change in bandgap is non-linear[1], with the bandgap increasing more slowly at higher strains. This trend correlates with the nonlinear increase of the internal parameter at higher strains. The inset iDPC-STEM images in Extended Data Fig. 3a confirm that the strained GaN lattice within the MiGs structure has a higher internal parameter. Extended Data Fig. 3b shows the calculated electronic band structures for both unstrained and -10% uniaxially strained GaN. Besides the increased bandgap, there is a shift in the ordering of the three valence bands, namely, the heavy hole (HH) band, light hole (LH) band, and split-off hole (SO) band. Notably, under high strain, the SO band becomes the topmost band, surpassing HH and LH by over 400 meV. This shift indicates a reversal of the crystal-field splitting energy sign compared to unstrained wurtzite GaN, where the SO band is below HH and LH bands. This valence band alteration significantly reduces the effective mass of holes along the *c*-direction. As illustrated in Extended Data Fig. 3b, the effective mass of split-off holes, proportional to the curvature of the parabolic band at the Γ-to-A direction, is much smaller than other bands. Consequently, uniaxially compressed strained GaN should exhibit significantly enhanced hole mobility along the *c*-direction due to the dominance of split-off hole bands and the small effective mass along Γ-to-A.

The STEM- CL spectra shown in Extended Data Fig. 3c compares cathodoluminescence from nano-scale sampling locations situated at varying distances from the MiGs structures, formed by annealing metallic Mg on an n-type GaN. The positions of these sampling locations are indicated in the HAADF-STEM image on the left, with a magnified STEM image confirming the presence of MiGs structures at locations #4 and #5. The resolution of this STEM image is compromised due to the thickness of the specimen required for STEM-CL measurement. The STEM-CL spectra

reveal a noticeable blue-shift of up to 10 nm in the positions of the peak near-band-edge (NBE) emissions, comparing areas containing MiGs (location #4) and the unstrained GaN within the bulk matrix phase (location #1). This blue-shift is smaller than the theoretically calculated value of approximately 13 nm, a discrepancy possibly attributable to the quantum-confined Stark effect (QCSE). The QCSE, resulting from the strong polarization field within the MiGs structure, likely causes a red shift in optical emission[2]. The collective redshift of the emissions from all the sampling positions could be a consequence of the high-energy Ga ion illumination on the FIB-prepared lamella specimen.

Extended Data Fig. 4 presents the plan-view and cross-sectional polarisation-resolved SEM-CL spectra of a p-type GaN both with and without MiGs nanostructures (Extended Data Fig. 4a). For plan-view CL spectra, we observed slight local variations in the degree of blue shift in the positions of the peak NBE emissions in MiGs-incorporated p-GaN (Extended Data Fig. 4c). These local variations seem to be independent of the surface morphology observed from secondary electron mode (Extended Data Fig. 4b), suggesting that they are more likely associated with a non-uniform distribution of MiGs phases. Similar to the STEM-CL spectra, the more blue-shifted points tend to exhibit further reduced NBE emission intensity. Compared to the blank p-GaN, the NBE emission becomes weaker in the MiGs-incorporated p-GaN, and the yellow luminescence (YL) intensifies compared to the blank p-GaN. The intensity of YL is less than one-tenth that of the NBE, suggesting that the predominant source of YL is the MiGs region. In contrast, the weaker NBE emission from the MiGs is thus overshadowed by emissions from other regions. Additionally, the YL band demonstrates a significant increase in transverse magnetic (TM) emissions, confirming that the emission from the MiGs is TM-polarized (Extended Data Fig. 4f). This observation aligns with the expected emission behaviour from the inter-band transition to the valence band, where split-off holes are dominant.

For further clarifications on the distinctions between STEM-CL and SEM-CL, two main aspects are considered: yellow luminescence and dopant concentration.

In the field of III nitride semiconductors, it is well-established that the origin of yellow luminescence in GaN is attributed to Ga vacancies or carbon impurities[3,4]. In STEM-based CL, the FIB-prepared sample undergoes high-energy ion illumination, leading to the creation of a large number of Ga vacancies, which in turn significantly increases the intensity of YL. As a result, across all effective sampling locations (#1-#4), the intensity of the peak YL is higher than that of the band-edge emission (see Extended Data Fig. 3d).

Conversely, in polarization-resolved SEM-based CL, the samples are not subjected to such ion illumination. Consequently, the intensity of the NBE emission peak is far greater than that of the YL (see Extended Data Fig. 4f).

Furthermore, the intensity ratio of YL to NBE emission differs significantly in samples before and after MiGs incorporation. The portion of YL is higher in samples with MiGs incorporation, both in STEM-CL (Extended Data Fig. 3d, location #4 compared to locations #1,#2) and in SEM-CL (Extended Data 4f). We believe that the enhanced YL can be attributed to a combination of factors. Firstly, the MiGs might contain higher levels of Ga vacancies (this could potentially be confirmed by positron annihilation spectroscopy with enhanced resolution[5]), which are due to the possible loss of Ga during the reaction with metallic Mg (charge transfer between Ga^{3+} to Mg^{0}). Secondly, aside from Ga vacancies, the presence of carbon impurities is also a contributing factor. As shown in the SIMS profiling (Supplementary Fig. S2), the concentration of O and C concentration in the Mg intercalated region is high, likely because the metallic Mg source is not pure, being only 99%-99.9% pure (up to three Ns). The high concentration of C may explain the higher ratio of YL to NBE emission in the MiGs compared to in the GaN. Thirdly, the enhanced YL might also be due to factors such as Mg doping, uneven distribution of MiGs structure, or the

impact of polarization. These factors could act as deep-level traps that lead to YL or non-radiative recombination centres.

Generally, setting aside other factors influence the CL intensity, the intensity of the CL signal in a homogeneous and defect-free sample is roughly inversely proportional to the dopant concentration, due to the reduced carrier diffusion length in a heavily doped sample. In our case, the n-type GaN sample has lower dopant concentration than p-type GaN sample. Therefore, the CL intensity should be weaker in the p-type GaN sample. Since the MiGs region exhibits weaker CL intensity, we anticipate that in SEM-CL, where the sampling volume is large, the collected signals could have a higher contribution ratio from MiGs-region in a p-type GaN.

The CL intensity is markedly weaker in regions containing MiGs structure, apart from being due to a possible higher carrier concentration, may also result from factors such as 2D-Mg_i doping, uneven distribution of MiGs structure, or the impact of polarization field[6,7]. Essentially, any mechanism that facilitates non-radiative recombination will decrease CL intensity. Since individual interstitial Mg (0D-Mg_i) introduces multiple deep-level traps, interstitial Mg intercalant sheets (2D-Mg_i) may create continuous deep-level traps, enhancing the non-radiative recombination of electrons and holes. A non-uniform distribution of MiGs structure might decrease CL intensity due to scattering at the domain boundary. Additionally, the impact of polarization could decrease CL intensity due to the strong polarization field and the possible gradient-induced hole doping by the polarization field. The latter factor actually increases carrier density and the strong electric field, as introduced previously. At this point, it is challenging to determine which factor(s) predominantly contribute to the decrease in CL intensities, as all could potentially lead to such a decrease. We believe that this topic warrants further in-depth study by designing multiple control samples.

Supplementary Note 2. Cross-sectional STEM observation of the MiGs morphologies on (0001) GaN

Following the deposition of metallic Mg onto (0001) GaN and subsequent annealing at elevated temperatures, the surface of GaN undergoes coarsening. Extended Data Fig. 5a-c displays cross-sectional view STEM images of the typical surface of as-annealed, post-cooled GaN (annealing condition: 800 °C, 10 min). The rough GaN surface can be observed. We also conducted this annealing experiment at a much lower temperature (450 °C, 10 min), where a slightly roughened surface was also observed. Furthermore, we noticed that the roughness scale increases with longer annealing times and higher temperatures.

A similar effect has not been well observed when Mg-containing compound are annealed on GaN. For example, despite attempts to anneal compounds like MgF_2 and MgO on GaN to improve ohmic contact, there have been no reported successes of such significant improvements. The difference likely stems from the chemical and electronic state of metallic Mg compared to these compounds. Metallic Mg (Mg^0) tends to lose electron in its metallic form, whereas it has already lost electrons as Mg^{2+} in MgF_2 and MgO. We hypothesize that the observed effects likely result from a chemical reaction between the alkaline earth metal Mg and GaN, inherently involving electron exchange. The electrons are probably transferred to Ga^{3+}. Given the significantly lower negative redox potential of Mg compared to Ga, a displacement reaction is assumed to be likely, indicating thermodynamic viability. This also potentially facilitates their incorporation into the GaN lattice. Consequently, we assumed that GaN could be chemically etched by Mg, akin to the etching process with KOH, with increased temperature and duration accelerating the reaction rate. As a result, finding evidence of Ga metal and Ga_2O_3 due to the oxidation of Ga^0 in air should be one of the future studies.

Additionally, as shown in Extended Data Fig. 5b, the surface layer contains remnants of amorphous Mg, along with MgO or possible magnesium nitride due to exposure to moisture, oxygen, or nitrogen at elevated temperatures. In addition, after cooling to room temperature and acid cleaning, we observed that the amorphous phases are removed (Extended Data Fig. 5d-h), indicating that they are residual amorphous Mg and Mg compounds that are soluble in acid.

Additionally, we noted that the upper continuous portion of the MiGs film tends to exfoliate, leaving pieces and voids on the surface (Extended Data Fig. 5e). This is likely due to the significant thermal mismatch between MiGs and the GaN matrix, as well as the extensive strain within the continuous MiGs film. Such exfoliation limits the achievable thickness and depth of MiGs, irrespective of longer annealing times or using thicker Mg films. In contrast, the lower triangular portion of MiGs, embedded deeper within the GaN matrix, tends to remain intact due to its discontinuous nature and reduced susceptibility to exfoliation (Extended Data Fig. 5g). Consequently, the MiGs phase does not form a continuous layer; an exfoliation line usually remains (as appeared in Extended Data Fig. 3c and Extended Data Fig. 5d, e), above which the continuous MiGs film is subject to several exfoliations, while below it, the discontinuous MiGs phases remain embedded in the GaN matrix (Extended Data Fig. 5a,d,f). These MiGs phases appear as giant inverted triangles, varying in size but mostly over 100 nm in diameter, scattered across the GaN surface (Extended Data Fig. 1 and Extended Data Fig. 5h).

The susceptibility of the continuous MiGs film to exfoliation limits the thickening of MiGs phase even with extending the annealing time. This is because that even if the MiGs layer becomes thicker, as depicted in Extended Data Fig. 1b, the thickened MiGs will have a continuous film portion at the upper part, which is prone to exfoliation during cooling and the subsequent acid cleaning process to remove remaining Mg compound. The remaining structure still predominantly

consists of a discontinuous MiGs phase with pyramidal domains (Extended Data Fig. 1c). Further efforts are necessary to devise methods to protect such structures from exfoliation.

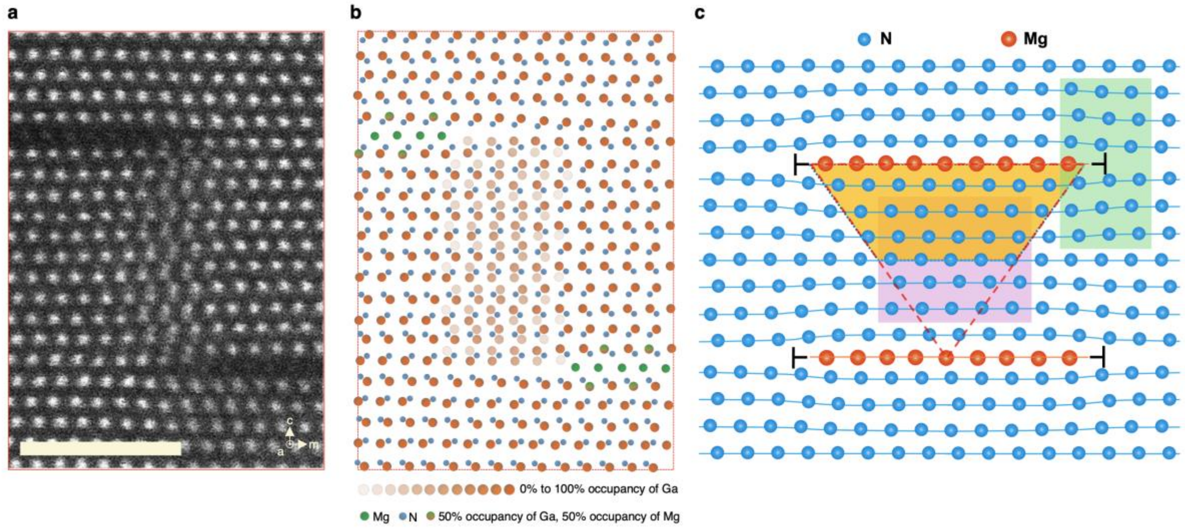

Supplementary Fig. S1 | Atomic arrangement around a pair of 2D-Mg_i sheets. a, A typical atomically resolved HAADF-STEM image showing lattice distortion at the tip of 2D-Mg_i sheets. Due to Z contrast, N and Mg are nearly invisible in this image, which is identical to Extended Data Fig. 5j. Scale bar: 2 nm. **b,** Schematic representation of the atomic arrangement in the same area as shown in a. The colour coding and labelling follow the same scheme as Fig. 2f in the Main section. **c,** Schematic representation of the arrangement of N and Mg atoms around a pair of 2D-Mg_i sheets (Ga atoms are omitted for simplicity, due to their polarity shift).

Regarding Extended Data Fig. 5i,j, (rearranged here as Supplementary Fig. S1a), we have depicted the schematic representation of the atomic arrangement in Fig. S1b. Since N atoms are not visible in ADF-STEM due to Z-contrast, the lattice distortion is attributed to Ga atoms. Interestingly, when using iDPC-STEM which visualize N atoms (Fig. 2a,b in Main section), we observed that the positions of N atoms largely remain unchanged, while Ga atoms exhibit a gradual shift from lower (N-polar) to upper (Ga-polar) sites from left to right. This shift is depicted with varying degrees of colour transparency to indicate the gradual change in the occupancy of Ga atoms at these sites (Fig. S1b).

We believe that measuring strain using drift-corrected HAADF-STEM images, based on the position of Ga atoms within the MiGs region (highlighted in purple area in Fig. S1c), is meaningful.

Conversely, the strain measured from the tip region in this way (highlighted in green area in Fig. S1c) appears to be excessively high, primarily due to a polarity shift in Ga atoms that results in a highly “distorted” appearance at the tip of the 2D-Mg_i sheet. However, considering that N atoms that remain unshifted and appear less “distorted”, as illustrated in Fig. S1b,c, the strain at the tip region of 2D-Mgi sheets should resemble to that observed at the core of edge dislocations (marked with the symbol ⊥ in Fig. S1c) [8], which is still excessive.

For the long-range interactions, it would be helpful to consider them as a displacement field in a continuum body. As illustrated in Fig. S1c, the two Mg sheets, acting as two vertically aligned half-edge dislocations of the same sign, exert a repelling force on each other. This repulsion is due to the long-range interactions of these two inserted half atomic planes; when they come too close to each other, the strain field increases.

On the other hand, due to the polarity transition of Ga, as the two Mg planes draw closer, fewer Ga atoms will undergo a polarity shift because the volume of the inversion domain shrinks (in Fig. S1c, the original inverted pyramidal shape delineated by red dotted line shrinks into the truncated pyramidal shape in yellow colour). This reduction in energy will lead to another set of long-range interactions, attracting the two sheets and aligning them along the *c*-axis. The observation of spontaneous alignment of these Mg sheets with typically 5-10 GaN layers between suggests that the energy reduction from shrinking the inversion domain outweighs the increase in the strain field. The balance between the attractive and repelling forces gives the long-range interactions within a pair of Mg sheets their characteristic features.

When 2D-Mg_i sheets form in pairs, the diameter of the upper sheet can significantly expand due to the “support” of the lower sheet, which truncates the shape of the pyramidal inversion domain induced by the upper sheet. The size expansion of these 2D-Mg sheets, resulting from the incorporation of additional interstitial Mg, resembles the climbing motion of edge dislocations.

This process leads to the consecutive expansion of vertically aligned 2D-Mg_i sheet arrays, evolving into a significantly large inverted pyramidal domain structure, with the bottommost Mg sheet forming a significantly small and untruncated pyramidal inversion domain.

Supplementary Note 3. Constraint of lattice swelling in interstitial intercalation

Since Mg sheets are orderly intercalated into every a few monolayers of GaN by the unique type of interstitial intercalation, a large uniaxial compressive strain is produced within the GaN layers. An inherent feature of interstitial intercalation is minimal lattice swelling. Here, a model assuming no lattice swelling, is proposed to estimate the elastic strain: N monolayers of GaN are intercalated with a pair of Mg sheets, then due to the continuity and integrity of the GaN layers and the feature of the interstitial intercalation, in the unintercalated GaN, the single interatomic spacing of *c*-planes is $\frac{c}{2}$,which is compressed into $\frac{c'}{2}$ by a pair of neighbouring Mg sheets. In addition, the spacing between the Mg layer and the nearest adjacent N layer is $\frac{c''}{2}$. N is the number of GaN monolayers being intercalated, and the spacings between neighbouring Mg sheet is denoted by $H_{\vec{c}}$. It is important to assume that $H_{\vec{c}}$ is conserved (i.e., no lattice swelling) after intercalation of Mg sheets, as depicted in the Supplementary Fig S2a, we have:

$$H_{\vec{c}} = (N-1) \times \frac{c}{2} + 2 \times \frac{c}{4} = (N-1) \times \frac{c'}{2} + 2 \times \frac{c''}{2} \quad \text{(S1)}$$

$$\varepsilon_c = \frac{c'-c}{c} = \frac{N}{N-1+2\eta} - 1 = \frac{1-2\eta}{N-1+2\eta} \quad \text{(S2)}$$

here the coefficient $\eta = \frac{c''}{c'}$ is introduced to associate c' with c''. The left term in Eqn. (S1) refers to the spacings outside the Mg intercalation region whereas the right term refers to the spacing inside the Mg intercalation region. The theoretically calculated ε_c is plotted in the Supplementary Fig. S2b with varying N (number of GaN monolayers). Similar to the internal parameter u (the

relative displacement between Ga and N sublattices along the c-axis), the coefficient η refers to the ratio of $\frac{c''}{2}$, which is the distance between Mg layer and the nearest N layer versus $\frac{c'}{2}$-the distance between neighbouring N layers. According to the model by John Northrup, the calculated $\frac{c''}{2}$ (distance between Mg layer and nearest N layer) is 2.22 Å, note that this is calculated under the condition of single monolayer Mg segregation, where uniaxial compressive strain is absent. Accordingly, in the unstrained GaN, $\frac{c'}{2}$ (distance between neighbouring N layers) takes the value of $\frac{c}{2} = \frac{5.189\,\text{Å}}{2} = 2.59\,\text{Å}$. Then η=2.22/2.59=0.857. However, under uniaxial compressive strain, both $\frac{c''}{2}$ and $\frac{c'}{2}$ are reduced due to compression in a slightly different degree, causing η to deviate from the unstrained value of 0.857. To avoid the complicated ab-initio calculation of the accurate change of $\frac{c''}{2}$ versus $\frac{c'}{2}$, we just assume a likely range of η 0.800 to 1.000 under which different uniaxial strain can be generated depending on the number of GaN monolayers being intercalated, according to Eqn. (S2).

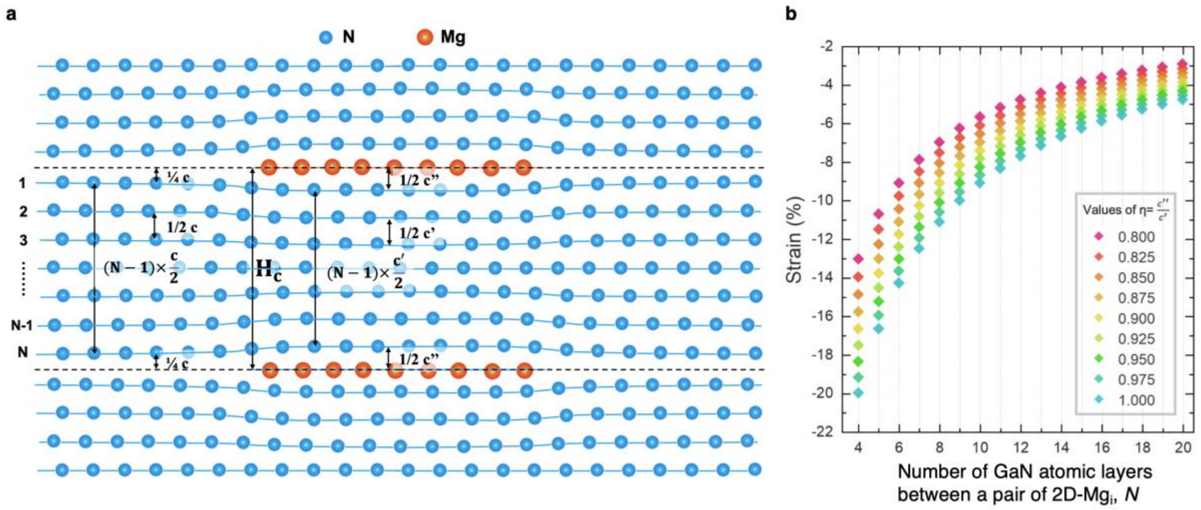

Supplementary Fig. S2 | Compressive elastic strain in GaN layers intercalated with Mg sheets. a, Schematic illustration of N monolayers GaN intercalated with a pair of Mg sheets assuming condition of non-lattice swelling. **b,** The relationship of uniaxial compressive strain versus the number of GaN atomic layers between a pair of 2D-Mg_i under different values of coefficient η.

Supplementary Note 4. SIMS investigation of the formation and development of MiGs in p-type GaN

Secondary Ion Mass Spectroscopy (SIMS) was employed to investigate the diffusion profile of Mg and other impurities (Si, O, C, and H) into epitaxial p-type GaN (Supplementary Fig. S3) in which Mg was initially substitutionally doped inside GaN by MOVPE (a process known as in-situ doping). In particular, the backside SIMS method was adopted, in which the samples were polished and thinned to ~1 μm before being sputtered from the backside during the SIMS measurement to circumvent the possible interference by the otherwise topmost Mg-intercalated GaN layer having rough surface and ultra-high Mg content. The Mg/GaN samples were annealed at different temperatures (500 °C, 600 °C, 700 °C, and 800 °C) for 10 min, followed by acid cleaning.

As shown in Supplementary Fig. S3, for all the p-type GaN samples being investigated, the SIMS profiles display a plateau of Mg around 6×10^{18} cm^{-3}, spanning approximately 400 nm. This suggests the characteristic of the initial p-type GaN before annealing with Mg. Notably, there is a significant increase in Mg concentration near the surface (60-100 nm) after annealing at 600 °C, 700 °C and 800 °C, indicating the formation of Mg intercalation and the enhanced Mg concentration in the MiGs-incorporated GaN.

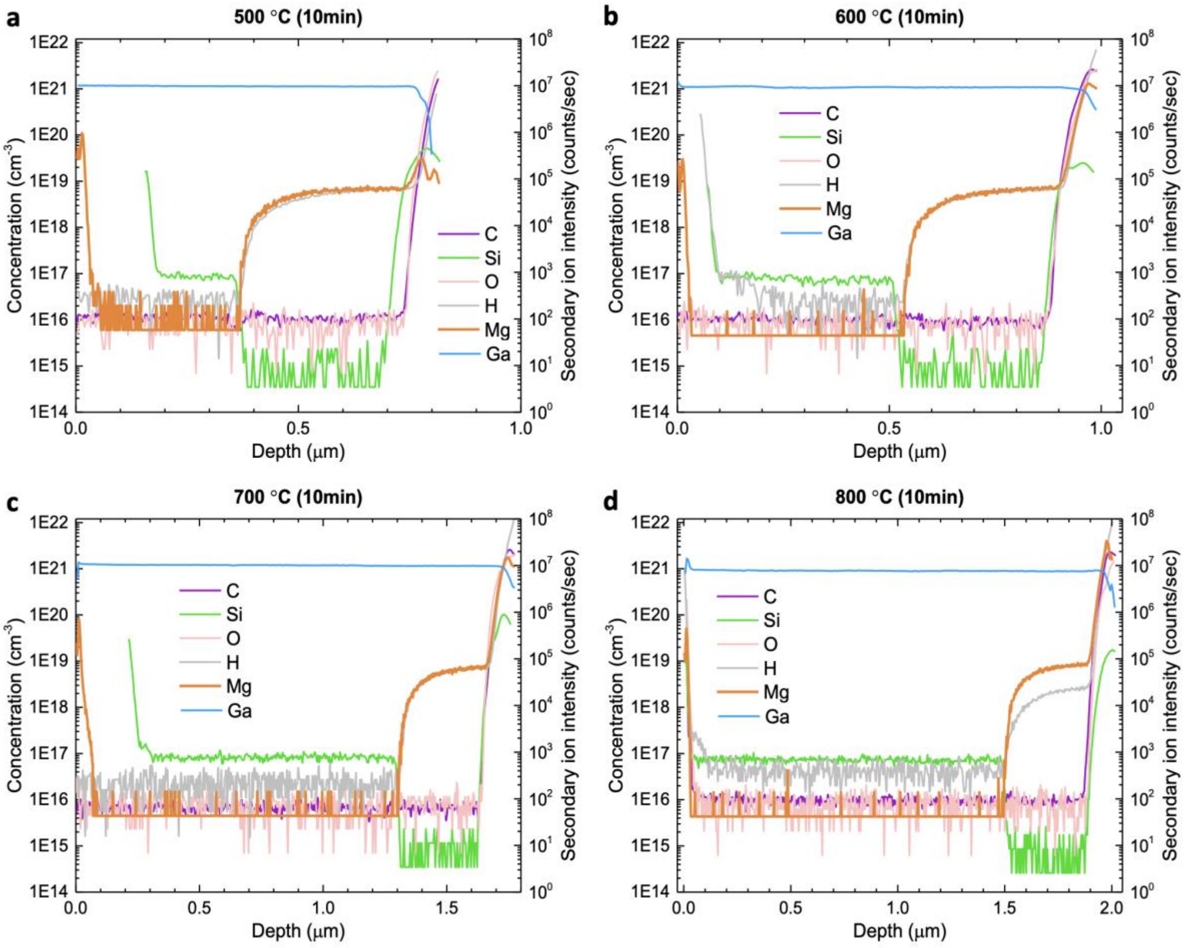

Supplementary Fig. S3 | Backside SIMS analysis of 400 nm-thick, uniformly Mg-doped p-type GaN samples with an initial Mg concentration of 6×10^{18} cm^{-3}, post-annealing of metallic Mg at varying temperatures followed by acid cleaning. a, 500 °C for 10 min. **b,** 600 °C for 10 min. **c,** 700 °C for 10 min. **b,** 800 °C for 10 min. The secondary ion intensity of Ga is displayed by the right vertical axis and the concentrations of C, Si, O, H, and Mg are displayed on the left vertical axis.

On the other hand, theoretically, the concentration of Mg in the MiGs can be calculated as follows:

$$[\mathrm{Mg}]_{\mathrm{MiGs}} = \frac{3}{\frac{3\sqrt{3}}{2}\times a'^2\times c'\times\left(\frac{N-1}{2}+\eta\right)} = \begin{cases} 7\times10^{21}\ \mathrm{cm^{-3}}\ (\text{for N} = 6) \\ 6\times10^{21}\ \mathrm{cm^{-3}}\ (\text{for N} = 7) \end{cases} \quad \text{(S3)}$$

Here, 3 indicates the number of Mg atoms in the hexagonal superlattice unit cell; a' and c' are lattice constants in the GaN layers, and η is the ratio of c'' to c'. N represents the number of GaN

monolayers intercalated by neighbouring Mg sheets. These symbols are referred to in Supplementary Fig. S2a.

Due to the nature of spontaneously formed MiGs, the spatial distribution of MiGs is not uniform after intercalation process and the inhomogeneous MiGs coexists with remaining unintercalated GaN. To find out the coverage Θ of MiGs in the mixture, we have:

$$\Theta(\mathrm{z}) = \frac{[\mathrm{Mg}]_{\mathrm{avg}}(\mathrm{z})}{[\mathrm{Mg}]_{\mathrm{MiGs}}} \quad (\text{assuming } [\mathrm{Mg}]_{\mathrm{MiGs}} \gg [\mathrm{Mg}]_{\text{unintercalated } p\text{-GaN}}) \tag{S4}$$

herein Θ is a function of depth z (assuming z=0 at the surface). Θ=1 suggests that MiGs covers the entire area whereas $\Theta \approx 0$ suggests that MiGs is almost negligible. As Mg diffuses downward into GaN, Θ reaches the maximum near the surface and gradually decreases along +z. According to Eqn. S4, the values of $\Theta(\mathrm{z}=0)$ for the samples annealed at different conditions are summarized in Supplementary Table 1. The peak concentration of [Mg] near the surface measured by SIMS is used as $[\mathrm{Mg}]_{\mathrm{avg}}(\mathrm{z}=0)$. For the condition of 500 °C, the maximum $\Theta \approx 0$ suggests that the formation of MiGs is not initiated, which agrees well with the HAADF-STEM observation. In stark contrast, the maximum coverage Θ increases significantly at 600 °C and higher temperatures, indicating enhanced interstitial diffusion of Mg into GaN in this temperature range.

Supplementary Table 1 | Maximum coverage of Mg-intercalated GaN at different temperatures

T (°C) for 10 min	$[\mathrm{Mg}]_{\mathrm{avg}}$ (z=0)	Θ (z=0) (6-ML-GaN)	Θ (z=0) (7-ML-GaN)
500	3.11×10^{19}	0.45%	0.52%
600	1.29×10^{21}	18.56%	21.64%
700	1.73×10^{21}	24.89%	29.03%
800	3.97×10^{21}	57.12%	66.61%

Supplementary Information References

1. Yan, Q., Rinke, P., Janotti, A., Scheffler, M. & Van de Walle, C. G. Effects of strain on the band structure of group-III nitrides. *Phys Rev B* **90**, 125118 (2014).
2. Leroux, M. *et al.* Quantum confined Stark effect due to built-in internal polarization fields in (Al, Ga) N/GaN quantum wells. *Phys Rev B* **58**, R13371 (1998).

3. Neugebauer, J. & Van de Walle, C. G. Gallium vacancies and the yellow luminescence in GaN. *Appl Phys Lett* **69**, 503–505 (1996).
4. Lyons, J. L., Janotti, A. & Van de Walle, C. G. Carbon impurities and the yellow luminescence in GaN. *Appl Phys Lett* **97**, (2010).
5. Armitage, R. *et al.* Contributions from gallium vacancies and carbon-related defects to the "yellow luminescence" in GaN. *Appl Phys Lett* **82**, 3457–3459 (2003).
6. Schroder, D. K. *Semiconductor Material and Device Characterization*. (John Wiley & Sons, 2015).
7. Lopatiuk-Tirpak, O. *et al.* Cathodoluminescence studies of carrier concentration dependence for the electron-irradiation effects in p-GaN. *Appl Phys Lett* **90**, (2007).
8. Zhao, C. W., Xing, Y. M., Zhou, C. E. & Bai, P. C. Experimental examination of displacement and strain fields in an edge dislocation core. *Acta Mater* **56**, 2570–2575 (2008).
